# Passive acoustics and sound recognition provide new insights on status and resilience of an iconic endangered marsupial (koala *Phascolarctos cinereus*) to timber harvesting

**DOI:** 10.1371/journal.pone.0205075

**Published:** 2018-10-31

**Authors:** Bradley S. Law, Traecey Brassil, Leroy Gonsalves, Paul Roe, Anthony Truskinger, Anna McConville

**Affiliations:** 1 Forest Science Unit, NSW Department of Primary Industries, Locked Bag 5123, Parramatta, NSW, Australia; 2 Science and Engineering Faculty, Queensland University of Technology, Brisbane, Australia; 3 EchoEcology, Crescent Head, NSW, Australia; University of Sydney, AUSTRALIA

## Abstract

Retention forestry aims to mitigate impacts of native forestry on biodiversity, but data are limited on its effectiveness for threatened species. We used acoustics to investigate the resilience of a folivorous marsupial, the koala *Phascolarctos cinereus*, to timber harvesting where a key mitigation practice is landscape exclusion of harvesting. We deployed acoustic recorders at 171 sites to record male bellows (~14,640 hours) for use in occupancy modelling and for comparisons of bellow rate (bellows night^-1^). Surveys targeted modelled medium-high quality habitat, with sites stratified by time since logging and logging intensity, including old growth as a reference. After scanning recordings with software to identify koala bellows, we found a high probability of detection (~0.45 per night), but this varied with minimum temperature and recorder type. Naïve occupancy was ~ 64% across a broad range of forests, which was at least five times more than expected based on previous surveys using alternative methods. After accounting for imperfect detection, probability of occupancy was influenced by elevation (-ve), cover of important browse trees (+ve), landscape NDVI (+ve) and extent of recent wildfire (-ve, but minor effect). Elevation was the most influential variable, though the relationship was non-linear and low occupancy was most common at tableland elevations (> 1000 m). Neither occupancy nor bellow rate were influenced by timber harvesting intensity, time since harvesting or local landscape extent of harvesting or old growth. Extrapolation of occupancy across modelled habitat indicates that the hinterland forests of north-east NSW support a widespread, though likely low density koala population that is considerably larger than previously estimated. Retention forestry has a significant role to play in mitigating harvesting impacts on biodiversity, including for forest specialists, but localised studies are needed to optimise prescriptions for koalas.

## Introduction

Globally, multiple use native forests produce pulp, timber, bioenergy and a range of other natural products. Unregulated forestry can result in environmental impacts and this has led to various modifications of silvicultural practices that provide for landscape exclusion zones and/or modified harvesting [1, 2, 3]. One model of forest management, referred to as ‘retention forestry’, aims to balance the goals of wood production and biodiversity conservation [4, 5]. Retention forestry retains single trees and/or intact forest patches at the time of harvest with the aim of conserving forest biodiversity and sustaining ecological functions [5]. A recent meta-analysis identified retention forestry as the most effective forest management approach in timber production forests for minimising species loss [6]. The area excluded from harvesting ranges from <5% to 40% of the local landscape, but there is little evidence to support what proportion is sufficient to maintain biodiversity values [4, 5, 7, 8].

Threatened species often have specialised requirements or greater sensitivity to disturbance regimes [5, 9, 10, 11]. For these species, additional mitigation measures are often employed to protect a proportion of feed trees, tree hollows, nectar resources or breeding sites, either as exclusion areas or as habitat elements within harvesting zones, but again little is known about the effectiveness of these more targeted management actions [7, 12]. Hence, determining their effectiveness remains a high priority [3, 13, 14].

The koala *Phascolarctos cinereus* is an iconic arboreal marsupial that occurs in widely varying densities in eucalypt forests and woodlands of eastern Australia [15]. The species is declining and is listed as vulnerable in a significant portion of its range [16]. Being an obligate folivore, koalas are associated with particular species of *Eucalyptus* that provide palatable foliage [17, 18]. The combined effect of environmental factors (e.g., topography, climate) and disturbances (e.g., fire) results in a spatially complex array of tree species within Australian eucalypt forests and, consequently, a mosaic of suitable and less suitable conditions for koalas [19]. Although mobile across highly modified landscapes [20, 21], koalas are impacted by permanent tree cover loss and fragmentation, as well as increased housing around bushland, road traffic, dog attack, climate change and disease (e.g., [16, 22, 23]).

Surveys in the north-east forests of New South Wales (NSW) have led to conclusions of a low likelihood of occurrence of koalas in the region [24]. For example, spot-lighting surveys detected koalas on 5% of 285 forest transects [25]. Similarly, call playback and spot-lighting detected koalas on 12% of 291 sites [26], while spot-lighting and scat searches targeting forestry areas recorded koalas on < 15% of sites [27]. A common problem with such surveys is imperfect detection (false absence–[28]), especially as koalas typically occur at low density and forests are not always easily accessible. Accounting for imperfect detection is possible in passive acoustic surveys that exploit male koala mating bellows, which are an advertisement call during the breeding season [29]. A preliminary passive acoustic survey in the north-east forests of NSW estimated higher than expected koala occupancy (~0.5 in higher quality habitats) [19]. Habitat modelling estimated 1.7 million ha of moderate to high quality koala habitat in this region (25% on the public timber production estate), suggesting the existence of a previously overlooked, but large population [19]. In this case, moderate to high quality habitat was derived from a MaxEnt model that classified the north-east forests on the basis of habitat suitability [19].

The influence of native forest timber harvesting on koalas is controversial and few studies have assessed direct impacts. Koalas tolerate a degree of habitat alteration following selective harvesting of shelter trees in the Pilliga forests, at least in the short term (i.e. six months after harvesting) [30]. A regional survey in forests of north-east NSW mostly recorded koalas in young (<30 years) regrowth, though at a low rate, and these regrowth areas were confounded with low elevation [26]. Moreover, koala scats are correlated positively with the number of selective harvesting events, indicating that koala populations are resilient to historical, low-intensity, harvesting [31, 32]. But scats are also associated with structurally complex, uneven-aged forests with some mature and old-growth elements, a large basal area and mixed species associations dominated by preferred browse species [32]. These results led [32] to suggest that high intensity timber harvesting that creates extensive gaps, especially those lacking mitigation measures, are incompatible with koala conservation. In the last 20 years, specific koala prescriptions have been implemented to protect koalas in forests harvested for timber, though they have relied upon detection of koala scat accumulations (high-use areas) during pre-harvest surveys [33].

Our study assessed koala occupancy and bellow rate across the extensive forested area of north-east NSW, Australia. We specifically targeted areas with different timber harvesting intensities and times since harvesting, as well as old growth forest. Harvesting ranged from recent modern practices where varied habitat prescriptions aim to mitigate environmental impact [7], to forests regenerating after historical harvesting that pre-dated standard environmental prescriptions. We also surveyed koala high-use areas that previous pre-harvest surveys identified based on scat accumulations, which then triggered a small ‘high-use’ exclusion area specifically for koalas. We surveyed koalas passively with acoustic recorders as these are effective for recording the male mating bellow during the spring breeding season [19, 29]. Occupancy and bellow rate were compared across harvesting treatments after first controlling for imperfect detection [34]. A second aim of our study was to provide an updated assessment of the status of koalas in the hinterland north-east forests of NSW based on occupancy estimates. Occupancy is an ideal population parameter for studies encompassing regional scales, especially as koalas typically occur at low densities [32, 34].

We predicted that if timber harvesting impacted koala populations, occupancy and calling rate would be greater in old growth forests and selectively harvested forests than heavily harvested forests, but that this impact would be moderated in areas with recently implemented prescriptions designed to protect koalas. We also predicted that occupancy and calling rate would increase with time since harvest.

## Materials and methods

### Study area

**Fig 1:**
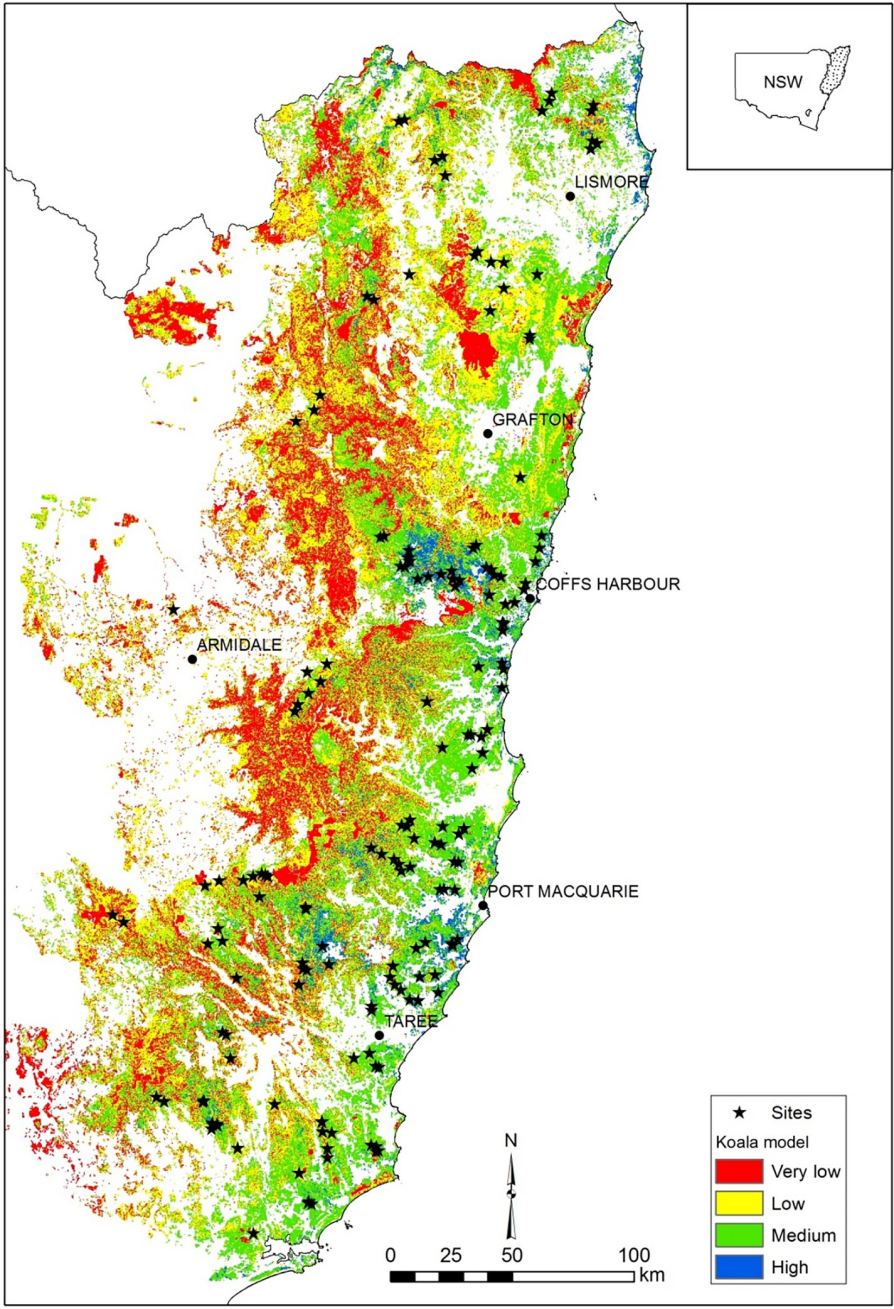
Location of 171 survey sites and distribution of modelled habitat used to target surveys in forests.

The study area spanned native forests of the hinterland, ranges and tablelands of north-east NSW bounded by the Hunter River in the south, the Queensland border in the north and Armidale in the west (~8.5 million ha) (Fig 1). The coastal strip was generally excluded as koalas in this area are exposed to multiple threats associated with urbanisation. Both State forests and National Parks were sampled, while private land (including private forestry) was excluded. The study region supports a rich diversity and mosaic of forest types, from tall moist eucalypt forest to dry sclerophyll forests and woodlands, including a range of important koala browse tree species [17, 35, 36]. A scientific license for our research was provided by the NSW national Parks and Wildlife Service (SL 102086). No animal ethics approval was required as only passive acoustic recording was undertaken.

### Survey design

Surveys targeted modelled medium-high quality koala habitat (MaxEnt model >0.45), which covers ~ 1.66 million ha of forest in north east NSW [19]. Thus forests of low predicted habitat quality and non-habitat (e.g. rainforest, heath) were excluded. We stratified our survey by time since harvesting and harvest intensity and sampled eight treatments (Table 1). Between 20–25 acoustic recorders (SongMeter SM2 or SM4, Wildlife Acoustics, Maynard, USA) were set each sampling trip, with one recorder located near the centre of an allocated treatment, though within a local landscape comprising a mosaic of forest ages and timber harvest exclusion zones. We sampled two levels of harvest intensity: light-moderate selective (<80 m^3^ timber removed per compartment (~ 250 ha)) and heavy harvesting (>80 m^3^ timber removed). Harvest intensity was designated based on recent Geographic Information System (GIS, ArcMap 10.4.1, ESRI) layers (GISO.EventPoly_State; FCNSW unpubl. data) or, for older forests (prior to 2001), using a Management History layer containing volumes of timber removed from compartments (FCNSW unpubl. data). Heavy harvesting, also referred to as regeneration harvesting, targeted dry hardwood forests, especially *E*. *pilularis* where site disturbance aids regeneration [37]. Within each harvest treatment, we sampled three different times since harvesting; recent (2–10 years since harvesting), medium (11–25 years since harvesting) and old (> 25 years since harvesting). Older harvested forests had few environmental protection measures at the time of harvest as environmentally sustainable practices had yet to develop [7]. In comparison, recent heavy harvesting incorporated exclusions for environmental protection, including for riparian zones, rainforest, old growth forest and specific protective measures for threatened species [7]. However, this treatment lacked any specific prescriptions for koalas because they were not previously detected by scat surveys. We also sampled koala high-use areas, which were areas where scat accumulations were detected by forestry surveys, and which were subsequently excluded from harvesting (in addition to other landscape exclusions). High-use areas are designated during pre-harvest surveys by either sighting a koala, identifying trees with > 20 scats or 30% of searched trees containing a scat [32]. Such areas were typically small patches (mean = 4 ha) with surrounding harvests occurring mostly < 10 years prior to acoustic sampling. Our final treatment was old growth forest, which was sampled for comparison with harvested sites. Old growth is mapped as a GIS layer (High Conservation Value Old Growth Forest) and although some of these sites had been lightly harvested historically, all sites represented long undisturbed forests. Within these treatments we sampled a range of topographic locations (gullies to ridge tops), forest types and elevations (10 to 1327 m above sea level (ASL)). Site locations were initially selected within GIS to sample the full range of forest treatments in each sampling session, and modified in the field if GIS mapping proved inaccurate. The mean minimum distance between sites was 5.6 km, which is considerably greater than the diameter of male koala home ranges (e.g. 60 ha) [21].

**Table 1:** Harvest treatments surveyed with acoustic recorders for koalas and number of replicate sites. Heavy harvests were defined as compartments treated by ‘heavy single tree selection’ or where more than 80 m^3^ per ha of timber were removed in an operation. Koala high-use areas were previously identified based on scat accumulations and resulted in patches (mean = 4 ha) being excluded from harvesting. All treatments were based on GIS mapped layers and field assessment. See S1 Table for treatment attributes.

Harvest Intensity	Time since harvest	Replicates
Heavy (>80 m^3^)	Recent (2–10 years)	24
	Medium(11–25 years)	6
	Old (>25 years)	21
Low-moderate (<80m^3^)	Recent (2–10 years)	28
	Medium(11–25 years)	24
	Old (>25 years)	30
Koala high-use/modified harvesting	< 15 years	14
Old growth	Little evidence of past harvesting	30

## Acoustic sampling

At each site, we deployed one Song Meter (SM2+ in 2015, SM4 in 2016/17– Wildlife Acoustics, Maynard USA) to record koala bellows. Song Meters were programmed to record from sunset until sunrise, the peak calling period of koalas [29], with a sampling rate of 22 kHz, and resolution of 16 bits per sample. We deployed a total of 171 Song Meters for at least seven consecutive nights (7–14 nights) over three breeding seasons (September-December) in each year between 2015 and 2017.

The distance at which koala calls can be detected is likely to vary with environmental conditions and topographic position. Studies using call playback at 75 dB in forests have found that call amplitude attenuates to background noise levels in recordings (e.g. SM2 recorders) within 100–150 m [38, 39], although we expect under ideal conditions and with later model acoustic recorders/microphones (e.g. SM4) this could extend to ~300 m based on detection distances for other species, such as owls [40]. [40] also noted that recording devices detect sounds over shorter distances than human ears and detection algorithms are less efficient for faint calls. Given variation in acoustic recorder models, background noise levels (e.g. during rain) and topographic locations of recorders, we assessed weather, topography and recorder unit effects on probability of detection and accounted for important effects in occupancy analyses (see below).

### Automated analysis of koala bellows

Recordings were scanned by acoustic software and a koala recogniser [41]. Recordings matched by the koala recogniser were checked for false positives by manually visualizing spectrograms of the audio and listening to recordings. Random checks were carried out for false negatives, revealing that very faint bellows were not recognised by the software. A single koala bellow comprised multiple event triggers. We defined a koala bellow as sequential event triggers that were <60 s apart. Elsewhere the mean length of a koala bellow has been reported as 36 sec and few last more than a minute [28, 42]. The number of koala bellows was tallied per site per night.

### Habitat and GIS covariates

A rapid assessment of habitat variables was undertaken within a 50 m radius around each recorder, which represents the central zone of detection of the recorder. Projected foliage cover of the canopy was measured using a smart phone application (‘Habitapp’ V1.1, Android application). Per cent cover was then apportioned based on a visual estimate to the different tree species comprising the canopy based on a visual estimate of their percentage contribution. Understorey cover was scored on the Braun Blanquet scale (<5%, 5–25%, 26–50%, 51–75%, > 75%) and the dominant contributor (rainforest, *Acacia*, vines, lantana, eucalypt regeneration) recorded. The presence of trees with hollows was scored as 1. common, 2. rare or 3. absent and stand structure was classed as 1. even or 2. uneven age. Harvest intensity and time since harvest were assessed from the presence of stumps and tree size to confirm the GIS classification of the site. Topographic position was scored on a scale of 1–12 (1 = summit, 12 = swamp). GIS was used to classify the surrounding landscape of each site within a 1 km buffer ([22]; Table 2) based on a number of GIS layers. This included the extent of wildfire and area harvested in the last 10 years, as well as the extent of old growth and cleared land (Table 2). We also derived site productivity by calculating normalized difference vegetation index (NDVI, [43]) values using MODIS MOD13Q1 granules acquired for September-December for each year between 2005 and 2015, at a 250 m scale and a 1 km site buffer.

**Table 2:** Description of covariates used to model variation in ρ (detectability) and ψ (occupancy).

Variable	Description
**ρ covariates**	
Month	Month of survey
Year	Year of survey (2015 –SM2, 2016 –SM4, 2016 –SM4)
Sampling effort	Number of sample nights site^-1^
Minimum temperature	Minimum temperature on survey night (^o^C) from nearest automated weather station
Nightly rainfall	Total rainfall during survey night (mm) from nearest automated weather station
Topographic position	Topographic position (upper, mid or lower slope)
Moon phase	Moon phase during survey
**ψ covariates**	
Harvest treatment	Timber harvest intensity and time since harvest, plus koala high-use and old growth
DEM^2^	Quadratic site elevation (m ASL)
Latitude	Latitude recorded for each site
Land tenure	State forest vs National park/reserve
Year	2015, 2016, 2017
Important browse	Projected foliage cover of class 1 and 2 tree browse species summed at each site (S2 Table)
Landscape recent harvesting	% area of recent harvesting (<10 years) in 1 km buffer
Landscape heavy harvesting	% area of recent heavy harvesting (<10 years) in 1 km buffer
Landscape old growth	% area of mapped old growth in 1 km buffer
Landscape cleared vegetation	% area of cleared land in 1 km buffer
Landscape wildfire	% area of recent wildfire (<10 years) in 1 km buffer
Landscape NDVI^2^	Quadratic NDVI^2^ value in 1 km buffer (spring value averaged over 10 years preceding survey)

### Occupancy modelling

We used single-season occupancy modelling to account for imperfect detection of koala bellows and to estimate probability of site occupancy [34]. We considered single-season occupancy to be appropriate for modelling because, although our study extended over three years, the low metabolic lifestyle of koalas, and the consequent restrictions on activity and reproduction [44], suggested little likelihood for significant change in populations over this short time frame. Moreover, each site was sampled in only one year and we included year as a covariate for occupancy in our modelling (see below). To restrict the number of models, we employed a hierarchical approach [45] whereby we first modelled probability of detection (ρ) from 7–14 consecutive nights of sampling while using a global model for occupancy that included all individual covariates for site occupancy (Ψ). Model selection was carried out using PRESENCE (V10.5, [46]) by comparing AIC scores of each model [47] to identify the most supported covariate for ρ, which was carried forward and used to model all subsequent parameters. Covariates used to model variation in ρ are listed in Table 2. Covariates modelled for Ψ included site-based disturbance (harvesting treatment, wildfire, clearing), habitat (foliage cover of important browse trees) and broad environmental (elevation, NDVI) variables, as well as landscape assessments of disturbance based on GIS layers (Table 1). Supported candidate models (dAIC < 2 points) were model-averaged to provide estimates of all parameters. All continuous covariates were standardised prior to analysis.

A Canonical Analysis of Principal coordinates (CAP) was conducted using PRIMER 6 (V6.1.18) and PERMANOVA+ (V1.0.8) (PRIMER-E Ltd, Plymouth, UK) to explore the relationship between different levels of occupancy and cover of individual tree species at each site, as well as supported covariates from modelling. Conditional occupancy for each site (probability that a site is occupied, given its particular detection history) was classified as probably absent (< 5%), low likelihood of occupancy (5–25%) and occupied (100%). The data were normalised and a Euclidean distance dissimilarity matrix was used.

## Results

Passive acoustic surveys were completed over three years at 171 sites resulting in ~14,640 hours of recording across 1,464 nights. Site and landscape attributes for each of the eight forest disturbance treatments are shown in S1 Table. Across the 171 sites, 2513 validated koala bellows were recorded at 62% of sites (naïve occupancy). On average, 1.6 bellows night^-1^ were recorded across the 171 sites surveyed. Koala high-use areas supported nearly three times the bellow rate (3.1 bellows night^-1^) as other treatments, but an ANCOVA found that the difference among treatments was not significant (F_7,162_ = 0.82; P = 0.6). Minimum nightly temperature was a significant covariate (-ve), indicating more frequent bellowing at lower temperatures (F_1,162_ = 5.93, P = 0.02). Bellow rate also did not vary with topographic position (F_1,162_ = 0.47, P = 0.62); i.e. among ridges, mid-slopes or gullies.

### Detection probability

In all, 40 candidate models were fitted for detection probability (S3 Table). Four models were supported, with model 1 allowing detection probability to vary with year and minimum nightly temperature, whereas models 2–4 also allowed detection to vary with nightly rainfall, moon phase and topographic position (Table 3).

**Table 3:** The top models (delta AIC < 2) fitted for ρ (detection probability) for koalas using songmeters to detect koala bellows.

Model	AIC	Delta AIC	AIC weight	Model likelihood	no. parameters	-2*log likelihood
ψ(global),ρ(yr+min temp)	1417.58	0.00	0.3994	1.0000	21	1375.58
ψ(global),ρ(yr+min temp+rain)	1418.51	0.93	0.2509	0.6281	22	1374.51
ψ(global),ρ(yr+min temp+moon.)	1419.20	1.62	0.1777	0.4449	22	1375.2
ψ(global),ρ(yr+min temp+topo)	1419.56	1.98	0.1484	0.3716	22	1375.56

**Fig 2:**
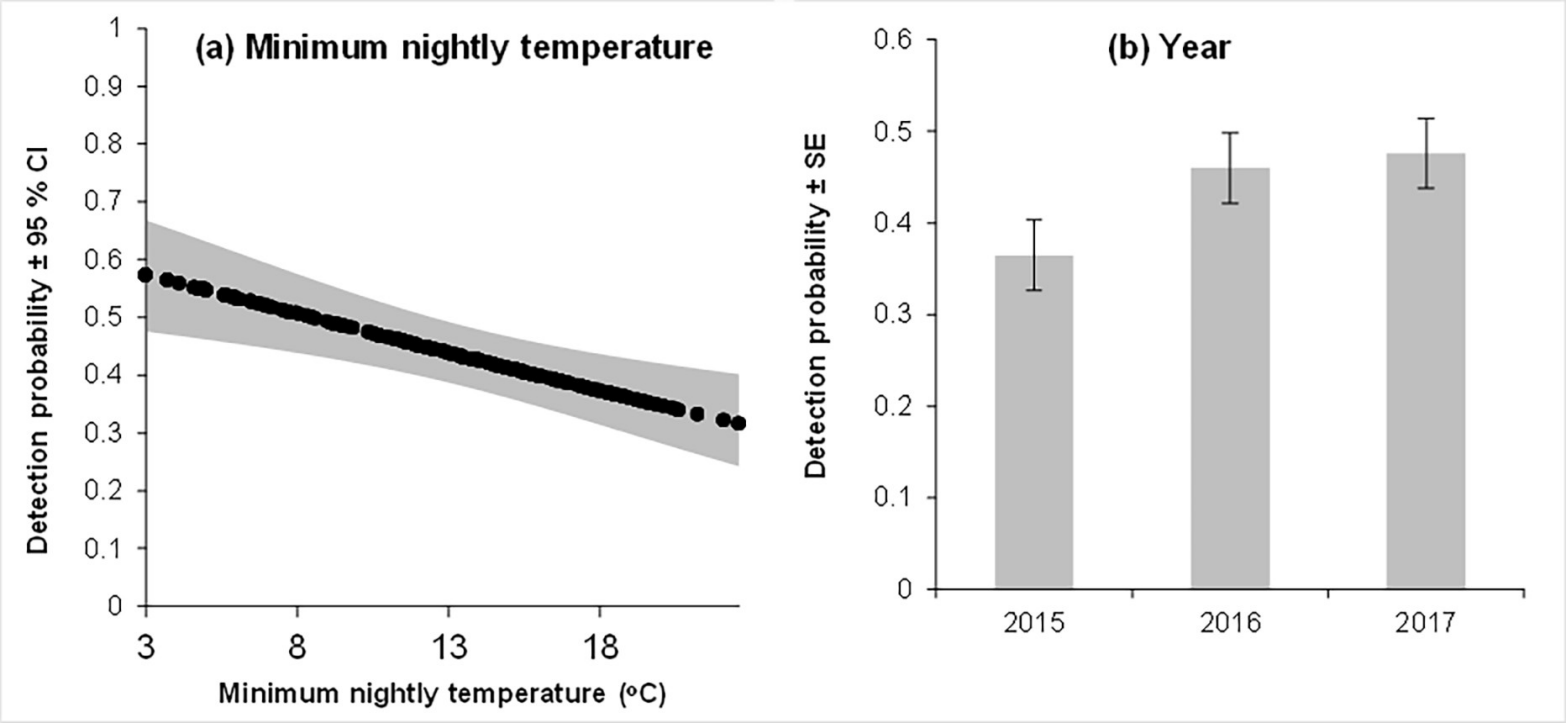
The relationship between modelled values of ρ (detection probability) for koalas and (A) minimum nightly temperature (when year is held constant) and (B) year (when minimum nightly temperature is held at its mean for each respective year).

Minimum nightly temperature had a major negative influence on detection probability (Fig 2). Detection probability declined from an estimated 0.57 per night at 3°C minimum temperature to 0.32 at 23°C. Minimum nightly temperature was also correlated negatively with month of survey, indicating lower detectability in December compared with September. Detection probability was also influenced by year of sampling, which was approximately 10% lower in 2015 (SM2 units) compared with 2016 and 2017 (SM4 units), respectively (Fig 2). The other covariates (influence of nightly rainfall (-ve), moon phase (-ve on full moons) and topographic position (+ve on ridges)) made only minor contributions to the models. For rainfall this is likely to be because little rain fell during the study.

### Occupancy

The covariates in the most supported model for detection probability (i.e., year and minimum nightly temperature) were carried forward to account for imperfect detection when modelling occupancy. In all, 35 candidate models were fitted (S2 Table). Forest treatment (trt) did not feature in any of the supported occupancy models (Table 4). For example, after holding other supported covariates at their mean, estimated occupancy across the full range of treatments, including old growth, was 0.64±0.04 (±SE). Similarly, landscape assessments of the extent of disturbance within the 1 km buffer did not influence occupancy. In addition, there was no support for occupancy to have been influenced by land tenure or to have changed over the three survey years.

**Table 4:** The top models (delta AIC < 2) fitted for probability of koala ψ (occupancy).

Model	AIC	Delta AIC	AIC weight	Model likelihood	no. of parameters	-2*log likelihood
ψ(DEM^2+feed trees),ρ(yr+min temp)	1403.07	0.00	0.1816	1.0000	8	1387.07
ψ(DEM^2+NDVI^2),ρ(yr+min temp)	1403.29	0.22	0.1626	0.8958	8	1387.29
ψ(DEM^2+fire),ρ(yr+min temp)	1403.69	0.62	0.1332	0.7334	8	1387.69
ψ(DEM^2*NDVI^2),ρ(yr+min temp)	1403.99	0.92	0.1146	0.6313	9	1385.99
ψ(DEM^2),ρ(yr+min temp)	1404.01	0.94	0.1135	0.6250	7	1390.01
ψ(DEM^2*feed trees),ρ(yr+min temp)	1405.06	1.99	0.0671	0.3697	9	1387.06

Modelling revealed support for six models with DEM^2^ included in each model (Table 2). Other supported covariates included as additive or interactive effects were important feed tree cover, NDVI^2^ and wildfire extent. The data were considered to be a reasonable fit to the supported models as assessed by the Pearson chi‐squared statistic (chi‐square = 61520.103, p = 0.06, ĉ = 1.8107).

**Fig 3:**
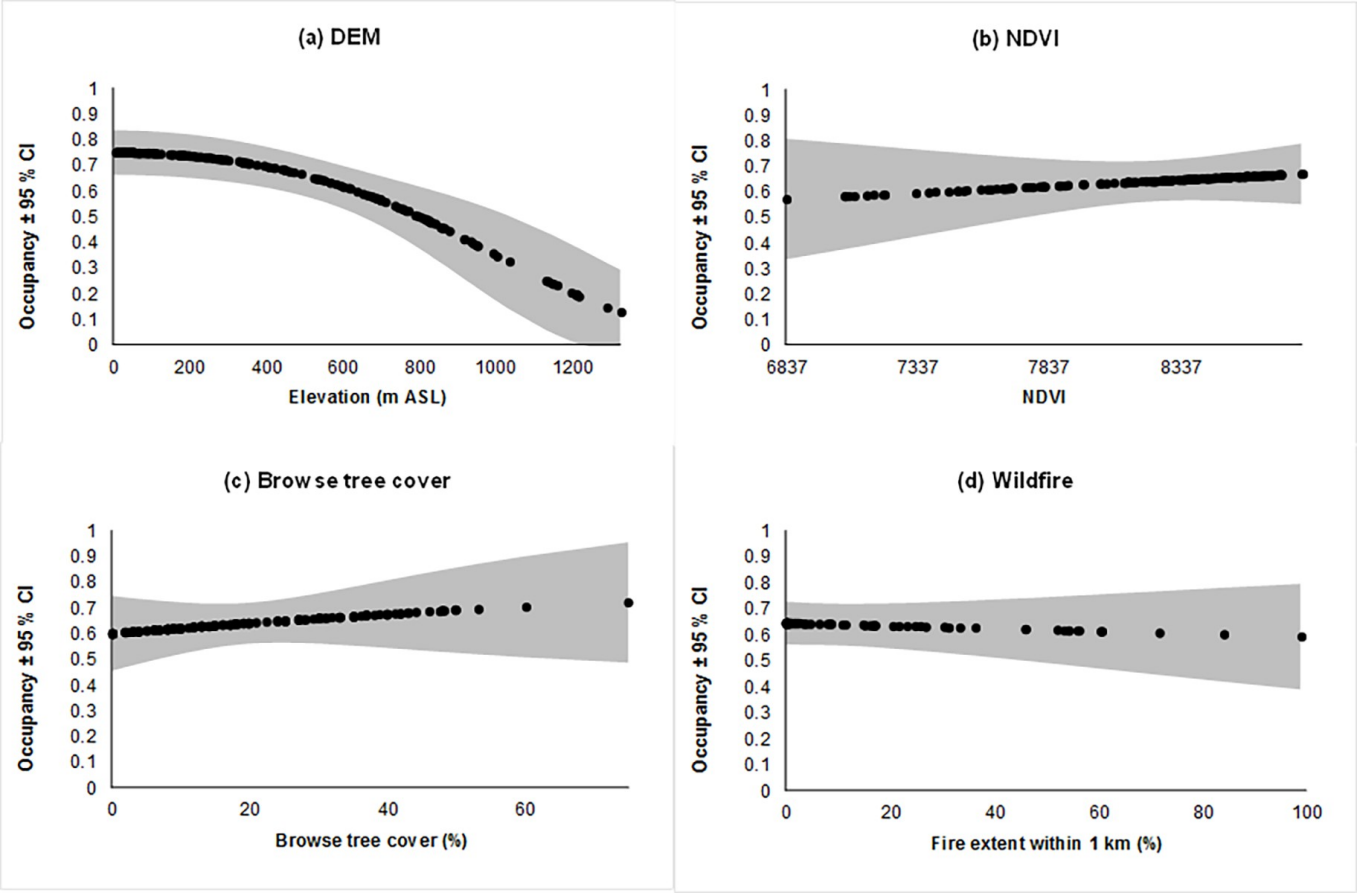
The relationship between modelled probability of occupancy for koalas and (A) elevation (quadratic), (B) NDVI (quadratic), (C) Important browse tree cover and (D) Wildfire extent in last 10 years. Other supported co-variates are held at their mean when displaying individual relationships. Grey areas indicate ±95% CLs.

Of the six supported models for koala occupancy, DEM^2^ showed the strongest association (negative) with occupancy, declining from an estimated occupancy of 0.75 at 10 m ASL to 0.13 at 1,327 m ASL (Fig 3). However, DEM^2^ plus the foliage cover of important browse trees had almost twice the AIC weight as DEM^2^ alone. All other associations (browse tree cover, NDVI^2^ and wildfire) were positive, though the strength of associations varied (Fig 3). Occupancy increased slightly (~10%) with both cover of browse species and NDVI. Occupancy was variable at low values of NDVI (generally from open forest in the Richmond River valley–e.g. Carwong S.F.), but was consistently higher at sites with high NDVI. Occupancy also declined slightly (~ 5%) with greater extent of wildfire, though we sampled few sites with a large wildfire extent.

**Fig 4:**
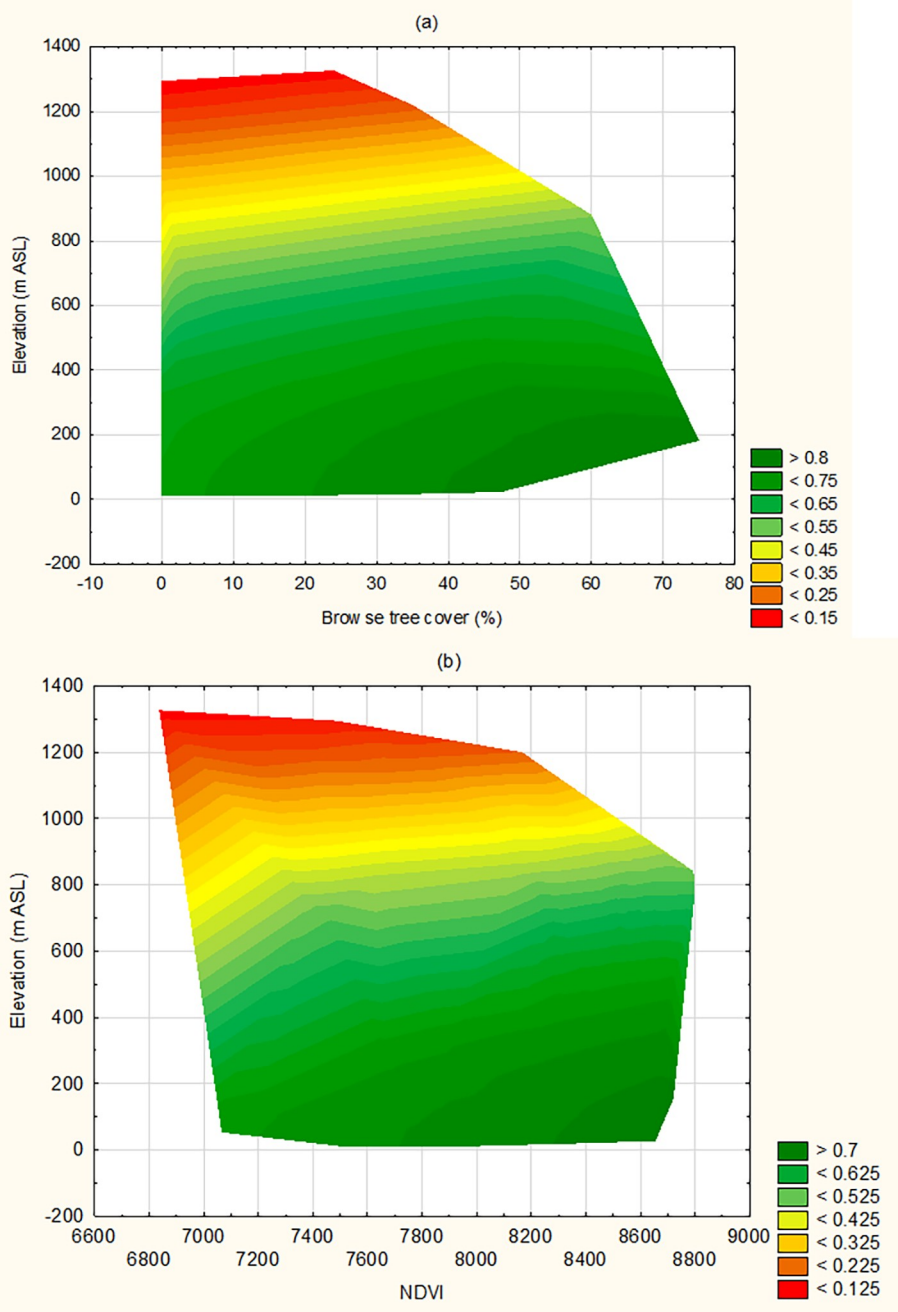
Wafer plot illustrating the interactive relationship on probability of occupancy for koalas for (A) Elevation by feed tree cover and (B) Elevation by NDVI while holding other supported covariates at their mean.

Two interactive effects were also supported in modelling, although neither were in the top model. The interaction of DEM^2^ and browse tree cover was such that probability of occupancy for koalas was high (>0.7) at low elevation even when there was low cover of important feed trees. As an example, some sites (e.g. Whian Whian SCA) had almost 100% cover of blackbutt *E*. *pilularis* (low importance browse tree), but high numbers of bellows. In contrast, at high elevation no sites were sampled with high cover of important browse trees, and occupancy was predicted to be close to zero when these were absent (Fig 4). Similarly, the interactive effect of NVDI^2^ and DEM^2^ indicated that at low elevations very low values of NVDI^2^ were not recorded, and that low-moderate values were associated with moderate koala occupancy (0.5–0.6) compared to negligible koala occupancy under both low and medium NDVI^2^ at high elevation (Fig 4).

### Koala browse species

**Fig 5:**
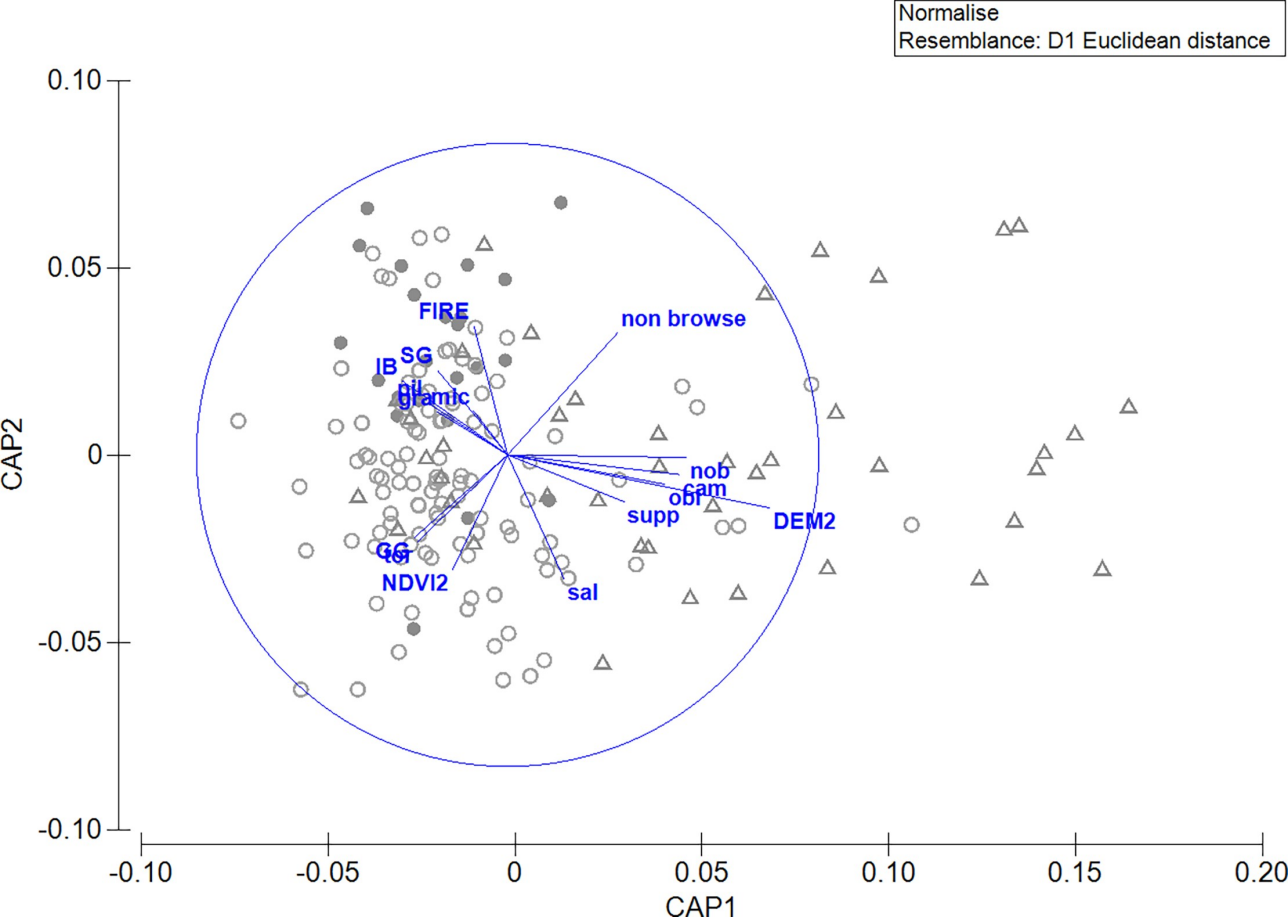
Canonical analysis of principal coordinates (CAP) illustrating site associations with environmental vectors (NDVI^2^, DEM^2^, common, ‘other’ important feed trees and non-browse species in study sites. Sites are grouped by conditional occupancy values (open circle = site occupied by koalas, closed circle = site with low likelihood (5–25%) of occurrence, and open triangle = site where koalas were likely absent (<5% probability of occurrence)). sal = *Eucalyptus saligna*, tor = *Allocasuarina torulosa*, gra = *Eucalyptus grandis*, mic = *Eucalyptus microcorys*, pil = *Eucalyptus pilularis*, GG = grey gum, IB = ironbark, SG = spotted gum, nob = *Eucalyptus nobilis*, obl = *Eucalyptus obliqua*, cam = *Eucalyptus campanulata*.

More than 42 tree species were identified at the survey sites (S2 Table). The most widely distributed tree species was tallowwood *Eucalyptus microcorys*, which is a primary browse species for koalas in the study region, being recorded at 120 sites, both occupied (75%) and likely absent sites (52%) (S2 Table). The CAP indicated that occupied sites were associated with higher cover of a wide range of tree species, such as *E*. *microcorys*, *E*. *saligna* and grey gum species, as well as important timber species (e.g., *E*. *pilularis*, ironbark and spotted gums) (Fig 5). Occupied sites were commonly at lower elevations and with greater NDVI^2^. Occupied sites at higher elevations were associated with the cover of ‘other’ browse and supplementary browse tree species (see also S2 Table). Sites where koalas were likely absent (<5% probability of occupancy) were associated with higher elevations (DEM^2^), low cover of important browse tree species and high cover of *E*. *nobilis*, *E*. *obliqua* and *E*. *campanulata* and other non-browse species (Fig 5). Sites with low likelihood of koala occupancy (5–25%) were associated with a greater extent of recent wildfire (within a 1 km buffer) and also more cover of spotted gum species (Fig 5; S2 Table).

## Discussion

Our study used innovative technology to automate identification of passively recorded koala calls and has provided new insights into the resilience of koalas to timber harvesting at a local landscape scale, as well as their status in hinterland forests of north-east NSW. Much higher detection rates (naïve occupancy) were recorded than previously known for the study region [25, 26]. After correcting for imperfect detection, occupancy did not vary with timber harvesting treatments, nor did bellow rate. Although we targeted modelled moderate-high quality koala habitat for our surveys, this represents an extensive area of north-east NSW, covering 1.66 million ha, approximately 25% of which is public timber production estate [19]. Koalas were broadly distributed throughout this area across many forest communities, comprising different browse tree species, and on the full range of topographic positions. Three covariates, and their interactions, were supported as influencing koala occupancy, including elevation, the cover of important browse trees and site productivity (NDVI). Extent of recent wildfire also had a minor influence on occupancy. Outside of modelled moderate to high quality habitat, a range of factors further reduce habitat quality for koalas such as high frequency wildfire, lack of browse species and low productivity soils on steep terrain [19].

### Bellow detection and interpretation of occupancy

Two primary covariates influenced koala detection probability. We found minimum nightly temperature had the greatest (negative) influence on detection probability and this was correlated with month of survey during the breeding season, with lower detectability in December than September. Similarly in Queensland, most male bellows are recorded during peak mating season in spring and there is a negative relationship with maximum nightly temperatures [29]. This pattern could reflect an energetic constraint of bellowing at warmer temperatures or bellow rate may simply reflect a correlation with the spring breeding period of koalas, where koalas call more frequently at the beginning of the season when temperatures are cooler and less at the end when temperatures are warmer [29]. We also found detection probability was influenced by year of sampling, being 10% lower in 2015 when SM2 units were deployed compared with 2016 and 2017 when SM4 units were used. SM2s were recently confirmed to have lower detectability and detection distances among a range of sound recorders tested due to less sensitive microphones [40]. Other covariates had a minor influence on bellow detection. We adjusted occupancy for the effects of variable detection probability (temperature and year/ acoustic recorder) and this is a key step needed when estimating occupancy.

Occupancy is an appropriate surrogate for population assessment when density is low as is expected for koalas [32, 35]. For example, given SM2/SM4s sample a 150–300 m radius of forest for koalas [38, 39], and male koala density in better quality forests of north-east NSW is 0.03 per ha [32], then just a single male would be expected in the acoustic sample area where a bellow was recorded. In addition to occupancy, bellow rate is correlated with koala density [39]. Bellowing also serves both to avoid male-male interactions by functioning as a signal of body size [48, 49] and to attract females [29], and thus an increase in bellow rate likely reflects an increase in breeding activity in a population [29, 50].

### Koalas and forest disturbance

Our study sampled a broad range of timber harvest intensities and times since harvesting, at both site (~300 m radius) and a larger landscape scale (1 km buffer), together with old growth forests for comparison. Neither occupancy nor bellow rate was found to be influenced by any of these treatments. These results are consistent with previous studies that have suggested koalas tolerate selective harvesting [26, 30, 32]. We also found occupancy and bellow rate were not lower in recent, heavily harvested forests after a significant component of the canopy had been removed. At the time of our surveys these sites were dominated by dense regeneration of sapling eucalypts in the understorey (mean = 5 years post-harvest). Although intensive harvesting in mixed species *E*. *pilularis* forests can favour the dominance of this species in the regeneration [36], koala occupancy remained high decades later in the old, heavy harvested stands, including those dominated by *E*. *pilularis*. Intensive harvesting of wet sclerophyll forests dominated by *E*. *microcorys* and *E*. *saligna* does not influence tree species diversity in the regenerating forest [51, 52].

Resilience of koalas to recent, heavy harvesting is most likely explained by the landscape mosaic of forest types and disturbance history in north-east NSW; especially the level of harvest exclusion in the landscape. Over the last 20 years exclusions averaged ~ 40% of the State forest area in the region [7]. In our study, about 50% of the 1 km area surrounding our recent, heavy harvest sites received this treatment in the last 10 years. The remainder comprised temporary off-set zones, but also permanent riparian buffers, old growth and rainforest exclusion areas and habitat protection for owls. In addition, large trees (40–80 cm dbh) provide important shelter and browse for koalas [31, 32]. Within the harvest area, scattered habitat trees, recruit/seed trees and feed trees for other species assist in providing a scattered uneven age structure, even where harvesting is heavy [7]. We recorded an average of 27% overstorey cover in recent, heavy harvest sites compared to 48% cover in old growth and old, heavy harvest sites (S2 Table).

Koala high-use exclusion areas represent one method of retaining patches (mean size = 4 ha) of large browse trees where an accumulation of koala scats had been identified prior to harvest, but we found occupancy was no greater in these areas than other treatments, and although bellow rate was greater, the difference was not significant. It is possible that retaining browse trees as clumps across a harvested zone might be more beneficial to koalas than focusing harvesting exclusion in a single area. Limited radio-tracking near Eden NSW has shown koala home ranges can comprise a mosaic of regrowth and unlogged habitat [53]. Foliage nutrient concentrations, including nitrogen, decrease with tree age in *E*. *grandis* and *E*. *pilularis* [54], and also tend to be higher in younger eucalypt foliage [55] or in foliage exposed to light [56]. In contrast, plant secondary metabolites are higher in foliage on small compared to large *E*. *microcorys* trees [36]. While this highlights the complex trade-offs between nutrients and toxins in browse resources, young trees, for example in plantations containing preferred browse species, can support high densities of koalas [20, 57]. More detailed fine scale movement data of koalas in a post-harvest landscape is needed to assess the effectiveness of different retention approaches, as well as assessing the extent to which koalas use young regenerating trees and exclusion areas post-harvesting.

Wildfire may have a greater immediate impact on koalas than timber harvesting both through direct mortality and indirectly by burning the canopy [58]. We found that the local extent of a wildfire in the last 10 years (usually a single fire) had only a small negative effect on occupancy. This is consistent with [21] who found that resource depletion from a major wildfire is short term for koalas because their mobility allows rapid recolonisation of the burnt forest, and they can maintain home ranges within forest regenerating from fire. Wildfire frequency rather than a single major fire may be a greater threat to koalas, noting that the areas we sampled had infrequent wildfires in the last 10 years. Indeed, wildfire frequency was a major contributor to modelling of koala habitat suitability in our study region [19].

### Key drivers of koala occupancy in NSW’s north-east forests

Rather than disturbance, the main drivers of koala occupancy were elevation, browse tree cover and landscape NDVI, as well as their interactions. Koala occupancy declined non-linearly with elevation and remained >0.5 at 700 m ASL, such as on the Dorrigo plateau. Lowest occupancy was found above 1,000 m ASL on the New England tablelands, where modelling of koala habitat also predicted high suitability areas to be scarce [19]. An association with low elevations has long been known (e.g., [17, 26, 59]); however, high occupancy at mid-elevation and even some high elevations (e.g., Nowendoc) appears to be less widely appreciated (but see [60, 61]). The New England Tablelands (and the north coast NSW) are predicted to provide climate refugia for koalas under climate change scenarios [62], but the interaction of elevation with important browse tree cover suggests many high elevation areas are unsuitable for koalas because less preferred browse species dominate (e.g. *E*. *nobilis*, *E*. *obliqua*, *E*. *campanulata*) and/or greater concentrations of plant secondary metabolites occur at higher, colder elevations [36]. While koalas were once considered to be “in great numbers” on the tablelands in the early 1900s [63], it is likely that many of the more suitable forests on fertile soils were preferentially cleared for agriculture and pine plantations.

Unsurprisingly, we also found occupancy increased with cover of important browse trees at a site. Koalas are obligate folivores that specialise on a small suite of eucalypts, though the use of these species may vary because of differences in site productivity or because the availability of desirable tree species varies between sites [17, 18, 64]. Browse tree selection at a local scale is complex and substantial differences are found in the amounts and types of chemical compounds in leaves of neighbouring *Eucalyptus* trees, even between trees of the same species [65]. We found an interaction between browse tree cover and elevation, which means occupancy was high at low elevation even where cover of important feed trees was low. At high elevation, occupancy was predicted to be almost zero when important feed trees were absent. This could mean that tree species not commonly recognised as important browse species are used more than currently expected at low elevations. Alternatively, low elevation sites with few important browse trees may be an artefact of our site-based assessment of browse tree cover in an area well known for its fine-scale mosaic of tree species and forest communities compared to that found at higher elevations [66]. Finally, koala occupancy also increased with NDVI, reflecting the importance of site productivity to browsers [43]. NDVI is also correlated with the presence and abundance of another eucalypt folivore, the greater glider *Petauroides volans* [67].

### Estimating the koala population in the north-east forests

Expert opinion currently suggests that the north coast and tablelands of north-east NSW supports a koala population of 8,367 (4,048–14,618) and 2,771 (468–5,838), respectively, though with high uncertainty (72–92% uncertainty) [15]. A minimum estimate based on our data for public-owned forests alone, suggests the population is substantially greater at more than 14,250 koalas (assuming a 1:1 sex ratio). We base this conservatively on just one male koala being present at occupied sites, a larger than expected sample area of 500 m radius surrounding acoustic recorders (78.5 ha), our established negative relationship with elevation and a range area of 0.83 million ha of predicted moderate to high quality koala habitat in public forests [19]. This estimate does not consider additional very low density koala populations in the extensive area of predicted low quality habitat. More importantly, it does not include privately-owned forests, which we did not survey, though they represent 50% of the predicted koala habitat for north-east NSW [19]. Systematic surveys are needed to estimate occupancy in private forests. We expect occupancy would be lower in many private forests than our estimate for public forests as they are often more fragmented by areas of permanent clearing. In particular, koalas in small forests near urbanisation are especially prone to direct mortality from cars and dogs [16, 22, 57, 68]. However, the value of coastal forests near urban centres, some of which are privately owned, should not be overlooked as they may support higher koala densities than hinterland forests when occurring on more fertile soils [32].

## Conclusion

In conclusion, koalas persist at high rates of occupancy and have a similar bellow rate across different timber harvest intensities and time since harvest in north-east NSW. Retention forestry likely plays a significant role in mitigating harvesting impacts on koalas, and other biodiversity [4, 5], but further research on movements would assist in revealing how exclusion areas and regeneration are used, and in optimising prescriptions for the species. In addition, passive recorders have revealed a widespread and large, but likely low density koala population in north-east NSW. This is consistent with the view that such populations have been poorly detected in less accessible parts of NSW and that koalas may be more resilient than suspected in those areas [69]. Our results also highlight that many National Parks and State forests are currently important custodians of koala populations in north-east NSW. The species continues to decline in many areas [16], but there are few data describing population trends. We suggest passive recorders have great potential for monitoring this and other low density populations of cryptic, but vocal animals.

## Supporting information

S1 Table Site attributes for eight different forest disturbance treatments. Treatments were classified by harvest intensity and time since harvest.
(DOCX)

S2 Table Tree species recorded in a 50 m radius around each acoustic recorder (n = 171 sites) in north-east NSW and their percentage occurrence at sites classified by conditional occupancy values of koalas. (DOCX)

S3 Table The candidate models fitted for detection probability and for probability of koala occupancy using acoustic recorders to detect koala bellows. (DOCX)
