# Supplementary material for: Passive acoustics and sound recognition provide new insights on status and resilience of an iconic endangered marsupial (koala Phascolarctos cinereus) to timber harvesting
Source: PLoS One. 2018 Oct 31;13(10):e0205075. doi: 10.1371/journal.pone.0205075 (PMC6209150; doi:10.1371/journal.pone.0205075)
Supplement: S1 Table — Treatments were classified by harvest intensity and time since harvest. (DOCX) [file pone.0205075.s001.docx]

Table S1: Site attributes for eight different forest disturbance treatments. Treatments were classified by harvest intensity and time since harvest. For GIS landscape variables, values are % of that variable in the surrounding 1 km buffer (mean ± SE). For NDVI (normalized difference vegetation index) values are the mean for a 250 m pixel in the 1 km buffer (mean ± SE). Site-based variables were assessed in a 50 m radius surrounding the Song Meter. Understorey cover is a visual estimate based on Braun Blanquet cover ranges (median for the treatment is reported), followed by the % occurrence of different understorey types (acacia, eucalypt regeneration, lantana, rainforest species, vines) in each treatment.

| Treatment | Heavy,<11 years | Heavy,11-24 years | Heavy,>24 years | Light-Medium,<11 years | Light-Medium,11-24 years | Light-Medium,>24 years | Koala High Use Area | Old Growth |
| --- | --- | --- | --- | --- | --- | --- | --- | --- |
| GIS derived Landscape variables (1 km radius) | | | | | | | | |
| Number of sites | 24 | 6 | 21 | 28 | 24 | 30 | 14 | 24 |
| Old growth (%) | 5 ± 2 | 1.1 ± 0.7 | 6.1 ± 1.9 | 4.2 ± 1 | 3.7 ± 1.2 | 7.9 ± 2.1 | 1.1 ± 0.6 | 30.5 ± 3.8 |
| Cleared (%) | 6.4 ± 1.9 | 15.4 ± 7.2 | 7.2 ± 1.7 | 10.7 ± 2.9 | 12.1 ± 2.9 | 6.5 ± 1.6 | 12 ± 3.4 | 6.8 ± 2.6 |
| Harvested  < 10 years (%) | 48.9 ± 4.2 | 10.1 ± 3.7 | 5.1 ± 2.9 | 37.1 ± 4.4 | 8.5 ± 2.9 | 5.8 ± 2 | 26.3 ± 5 | 3.5 ± 1.4 |
| Heavy harvest < 10 years (%) | 41.6 ± 4.1 | 0 | 0.6 ± 0.4 | 2 ± 0.9 | 0.9 ± 0.7 | 0.7 ± 0.5 | 11.5 ± 4.7 | 1.5 ± 1 |
| Wildfire < 10 years (%) | 8.5 ± 4.2 | 0.3 ± 0.2 | 12.8 ± 6.1 | 10.7 ± 3.9 | 4 ± 2.3 | 3.3 ± 1.4 | 10.7 ± 5.3 | 5.7 ± 2.5 |
| NDVI (average) | 8160 ± 69 | 8274 ± 228 | 8435 ± 54 | 8092 ± 91 | 8061 ± 113 | 8246 ± 65 | 8102 ± 129 | 8325 ± 87 |
| Elevation (m) | 312 ± 76.9 | 120.5 ± 59.4 | 484.2 ± 78.5 | 503.7 ± 74.9 | 437.5 ± 79.5 | 385 ± 59.6 | 324.9 ± 70.5 | 491.3 ± 74.9 |
| Site habitat variables (50 m radius) | | | | | | | | |
| Hollows (% sites) |  |  |  |  |  |  |  |  |
| absent | 55 | 100 | 33 | 55 | 50 | 38 | 38 | 0 |
| rare | 45 | 0 | 67 | 40 | 50 | 57 | 62 | 29 |
| common | 0 | 0 | 0 | 5 | 0 | 5 | 0 | 71 |
| Upper stratum Cover (PFC%) | 27 ± 3 (24) | 45 ± 6 (6) | 48 ± 1 (21) | 29 ± 3 (27) | 39 ± 2 (23) | 44 ± 2 (29) | 46 ± 4 (14) | 48 ± 2 (24) |
| Uneven stand age  (% sites) | 86 | 0 | 60 | 85 | 100 | 74 | 92 | 100 |
| Understorey cover  (% cover) | 51-75 | 51-75 | 26-50 | 51-75 | 5-25 | 51-75 | 26-50 | 51-75 |
| Acacia  (% sites) | 45 | 25 | 27 | 55 | 57 | 30 | 38 | 19 |
| Eucalypt regeneration (% sites) | 100 |  | 20 | 90 | 71 | 35 | 62 | 24 |
| Lantana (% sites) | 18 | 25 | 7 | 5 | 14 | 4 | 23 | 5 |
| Rainforest species (% sites) | 14 | 75 | 60 | 20 | 21 | 52 | 31 | 62 |
| Vines (% sites) | 5 |  | 7 | 15 | 21 | 9 | 8 | 29 |
