# Supplementary material for: Passive acoustics and sound recognition provide new insights on status and resilience of an iconic endangered marsupial (koala Phascolarctos cinereus) to timber harvesting
Source: PLoS One. 2018 Oct 31;13(10):e0205075. doi: 10.1371/journal.pone.0205075 (PMC6209150; doi:10.1371/journal.pone.0205075)
Supplement: S2 Table — (DOCX) [file pone.0205075.s002.docx]

Table S2: Tree species recorded in a 50 m radius around each Song Meter (n= 171 sites) in north-east NSW and their percentage occurrence at sites classified by conditional occupancy values of koalas. Occupied = Percentage of occupied sites where the tree species was recorded; Low occupancy (5-25 % probability of occupancy) = Percentage of low occupancy sites where the tree species was recorded; Absent (< 5 % probability of occupancy) = Percentage of sites without koalas, where the tree species was recorded. Number of sites = Total number of sites where the browse species was recorded. Browse category is a classification of tree species in 4 classes of browse tree preference for koalas from 1 (most preferred) to 4 (least preferred). Where common names are listed the precise tree species was not identified in the field.

| Tree Species | Browse category | Number of sites | Occupied | Low occupancy | Absent |
| --- | --- | --- | --- | --- | --- |
| *Eucalyptus microcorys* | 1 | 120 | 74.5 | 82.6 | 52.4 |
| *Eucalyptus saligna* | 2 | 71 | 43.4 | 17.4 | 50 |
| *Eucalyptus pilularis* | 3 | 60 | 40.6 | 43.5 | 16.7 |
| *Allocasuarina torulosa* | 2 | 44 | 32.1 | 26.1 | 9.5 |
| Ironbark | 3 | 31 | 24.5 | 13 | 4.8 |
| Grey Gum | 2 | 30 | 22.6 | 8.7 | 9.5 |
| Mahogany | 3 | 26 | 18.9 | 4.3 | 11.9 |
| *Corymbia intermedia* | 4 | 22 | 17 | 13 | 2.4 |
| *Eucalyptus propinqua* | 2 | 27 | 15.1 | 39.1 | 4.8 |
| Stringybark | 3 | 25 | 13.2 | 4.3 | 23.8 |
| *Eucalyptus grandis* | 2 | 16 | 12.3 | 13 | . |
| *Corymbia maculata* | 4 | 18 | 11.3 | 13 | 7.1 |
| *Eucalyptus acmenoides* | 3 | 21 | 10.4 | 30.4 | 7.1 |
| *Eucalyptus paniculata* | 3 | 13 | 7.5 | 17.4 | 2.4 |
| *Eucalyptus campanulata* | 4 | 18 | 5.7 | . | 28.6 |
| *Eucalyptus tereticornis* | 1 | 6 | 5.7 | . | . |
| *Syncarpia glomulifera* | 4 | 14 | 4.7 | 39.1 | . |
| *Corymbia variegata* | 4 | 6 | 3.8 | 8.7 | . |
| *Eucalyptus laevopinea* | 3 | 9 | 3.8 | . | 11.9 |
| *Eucalyptus moluccana* | 2 | 6 | 3.8 | 8.7 | . |
| *Eucalyptus punctata* | 2 | 4 | 3.8 | . | . |
| *Lophostemon confertus* | 4 | 10 | 3.8 | 13 | 7.1 |
| *Allocasuarina littoralis* | 3 | 4 | 2.8 | 4.3 | . |
| *Corymbia gummifera* | 4 | 8 | 2.8 | 17.4 | 2.4 |
| *Eucalyptus siderophloia* | 3 | 8 | 2.8 | 21.7 | . |
| Rainforest spp. | 4 | 9 | 2.8 | 4.3 | 11.9 |
| Red gum | 1 | 7 | 2.8 | 17.4 | . |
| *Eucalyptus largeana* | 2 | 2 | 1.9 | . | . |
| *Eucalyptus nobilis* | 3 | 10 | 1.9 | . | 19 |
| *Eucalyptus radiata* | 2 | 4 | 1.9 | . | 4.8 |
| Scribbly gum | 3 | 3 | 1.9 | 4.3 | . |
| Box | 3 | 1 | 0.9 | . | . |
| Eucalypt sp. | 3 | 4 | 0.9 | 4.3 | 4.8 |
| *Eucalyptus biturbinata* | 2 | 2 | 0.9 | . | 2.4 |
| *Eucalyptus obliqua* | 3 | 10 | 0.9 | . | 21.4 |
| *Eucalyptus resinifera* | 3 | 5 | 0.9 | 17.4 | . |
| *Eucalyptus seeana* | 2 | 1 | 0.9 | . | . |
| Peppermint | 3 | 1 | 0.9 | . | . |
| *Angophora costata* | 4 | 1 | . | 4.3 | . |
| *Araucaria cunninghamii* | 4 | 1 | . | 4.3 | . |
| *Eucalyptus cameronii* | 3 | 2 | . | . | 4.8 |
| *Eucalyptus deanei* | 3 | 1 | . | . | 2.4 |
| *Eucalyptus fastigata* | 3 | 2 | . | . | 4.8 |
| *Eucalyptus fibrosa* | 3 | 1 | . | 4.3 | . |
| *Eucalyptus globoidea* | 3 | 1 | . | 4.3 | . |
| *Eucalyptus quadrangulata* | 3 | 1 | . | . | 2.4 |
| *Eucalyptus robusta* | 1 | 1 | . | 4.3 | . |
| *Eucalyptus viminalis* | 1 | 2 | . | . | 4.8 |
| Gum | 3 | 1 | . | . | 2.4 |
| *Lophostemon suaveolens* | 4 | 1 | . | 4.3 | . |
| *Melaleuca quinquenervia* | 4 | 1 | . | 4.3 | . |
| She-Oak | 3 | 2 | . | 8.7 | . |
| Spotted gum | 4 | 2 | . | 8.7 | . |
