# Supplementary material for: Passive acoustics and sound recognition provide new insights on status and resilience of an iconic endangered marsupial (koala Phascolarctos cinereus) to timber harvesting
Source: PLoS One. 2018 Oct 31;13(10):e0205075. doi: 10.1371/journal.pone.0205075 (PMC6209150; doi:10.1371/journal.pone.0205075)
Supplement: S3 Table — (DOCX) [file pone.0205075.s003.docx]

**Table S3a. The 40 candidate models fitted for detection probability for koalas using acoustic recorders to detect koala bellows.**

| **Model** | **AIC** | **Delta AIC** | **AIC weight** | **Model likelihood** | **no. of parameters** | **-2*log likelihood** |
| --- | --- | --- | --- | --- | --- | --- |
| ψ(global),ρ(yr+min temp) | 1417.58 | 0.00 | 0.3994 | 1.0000 | 21 | 1375.58 |
| ψ(global),ρ(yr+min temp+rain) | 1418.51 | 0.93 | 0.2509 | 0.6281 | 22 | 1374.51 |
| ψ(global),ρ(yr+min temp+moon.) | 1419.20 | 1.62 | 0.1777 | 0.4449 | 22 | 1375.2 |
| ψ(global),ρ(yr+min temp+topo) | 1419.56 | 1.98 | 0.1484 | 0.3716 | 22 | 1375.56 |
| ψ(global),ρ(yr) | 1426.51 | 8.93 | 0.0046 | 0.0115 | 20 | 1386.51 |
| ψ(global),ρ(yr+rain) | 1426.78 | 9.20 | 0.0040 | 0.0101 | 21 | 1384.78 |
| ψ(global),ρ(yr+moon) | 1427.42 | 9.84 | 0.0029 | 0.0073 | 21 | 1385.42 |
| ψ(global),ρ(yr+rain+moon) | 1427.92 | 10.34 | 0.0023 | 0.0057 | 22 | 1383.92 |
| ψ(global),ρ(month+yr) | 1428.27 | 10.69 | 0.0019 | 0.0048 | 21 | 1386.27 |
| ψ(global),ρ(yr+topo) | 1428.47 | 10.89 | 0.0017 | 0.0043 | 21 | 1386.47 |
| ψ(global),ρ(month+yr+rain) | 1428.53 | 10.95 | 0.0017 | 0.0042 | 22 | 1384.53 |
| ψ(global),ρ(yr+rain+topo) | 1428.69 | 11.11 | 0.0015 | 0.0039 | 22 | 1384.69 |
| ψ(global),ρ(month+yr+moon) | 1429.31 | 11.73 | 0.0011 | 0.0028 | 22 | 1385.31 |
| ψ(global),ρ(yr+moon+topo) | 1429.36 | 11.78 | 0.0011 | 0.0028 | 22 | 1385.36 |
| ψ(global),ρ(month+yr+topo) | 1430.25 | 12.67 | 0.0007 | 0.0018 | 22 | 1386.25 |
| ψ(global),ρ(sample nights) | 1438.77 | 21.19 | 0.0000 | 0.0000 | 18 | 1402.77 |
| ψ(global),ρ(min temp+moon) | 1448.24 | 30.66 | 0.0000 | 0.0000 | 19 | 1410.24 |
| ψ(global),ρ(min temp+rain+moon) | 1448.90 | 31.32 | 0.0000 | 0.0000 | 20 | 1408.9 |
| ψ(global),ρ(min temp) | 1449.44 | 31.86 | 0.0000 | 0.0000 | 18 | 1413.44 |
| ψ(global),ρ(min temp+moon+topo) | 1449.59 | 32.01 | 0.0000 | 0.0000 | 20 | 1409.59 |
| ψ(global),ρ(min temp+rain) | 1450.24 | 32.66 | 0.0000 | 0.0000 | 19 | 1412.24 |
| ψ(global),ρ(min temp+topo) | 1450.44 | 32.86 | 0.0000 | 0.0000 | 19 | 1412.44 |
| ψ(global),ρ(month+min temp) | 1450.74 | 33.16 | 0.0000 | 0.0000 | 19 | 1412.74 |
| ψ(global),ρ(min temp+rain+topo) | 1451.05 | 33.47 | 0.0000 | 0.0000 | 20 | 1411.05 |
| ψ(global),ρ(month+rain+moon) | 1461.94 | 44.36 | 0.0000 | 0.0000 | 20 | 1421.94 |
| ψ(global),ρ(month+rain) | 1463.00 | 45.42 | 0.0000 | 0.0000 | 19 | 1425 |
| ψ(global),ρ(month+moon) | 1463.17 | 45.59 | 0.0000 | 0.0000 | 19 | 1425.17 |
| ψ(global),ρ(month+rain+topo) | 1463.58 | 46.00 | 0.0000 | 0.0000 | 20 | 1423.58 |
| ψ(global),ρ(month) | 1463.97 | 46.39 | 0.0000 | 0.0000 | 18 | 1427.97 |
| ψ(global),ρ(month+moon+topo) | 1464.41 | 46.83 | 0.0000 | 0.0000 | 20 | 1424.41 |
| ψ(global),ρ(month+topo) | 1464.88 | 47.30 | 0.0000 | 0.0000 | 19 | 1426.88 |
| ψ(global),ρ(rain+moon+topo) | 1471.60 | 54.02 | 0.0000 | 0.0000 | 20 | 1431.6 |
| ψ(global),ρ(rain+moon) | 1472.12 | 54.54 | 0.0000 | 0.0000 | 19 | 1434.12 |
| ψ(global),ρ(rain+topo) | 1472.59 | 55.01 | 0.0000 | 0.0000 | 19 | 1434.59 |
| ψ(global),ρ(rain) | 1473.78 | 56.20 | 0.0000 | 0.0000 | 18 | 1437.78 |
| ψ(global),ρ(moon+topo) | 1474.53 | 56.95 | 0.0000 | 0.0000 | 19 | 1436.53 |
| ψ(global),ρ(moon) | 1474.65 | 57.07 | 0.0000 | 0.0000 | 18 | 1438.65 |
| ψ(global),ρ(topo) | 1475.27 | 57.69 | 0.0000 | 0.0000 | 18 | 1439.27 |
| ψ(global),ρ(.) | 1476.00 | 58.42 | 0.0000 | 0.0000 | 17 | 1442 |
| ψ(global),ρ(trt) | 1476.76 | 59.18 | 0.0000 | 0.0000 | 24 | 1428.76 |

Dark grey shading indicates supported models for detectability. Light grey shading indicates the most supported model for detection which was carried forward when modelling koala occupancy.

**Table S3b. The 35 candidate models fitted for probability of koala occupancy.**

| **Model** | **AIC** | **Delta AIC** | **AIC weight** | **Model likelihood** | **no. of parameters** | **-2*log likelihood** |
| --- | --- | --- | --- | --- | --- | --- |
| ψ(DEM^2+feed trees),ρ(yr+min temp) | 1403.07 | 0.00 | 0.1816 | 1.0000 | 8 | 1387.07 |
| ψ(DEM^2+NDVI^2),ρ(yr+min temp) | 1403.29 | 0.22 | 0.1626 | 0.8958 | 8 | 1387.29 |
| ψ(DEM^2+fire),ρ(yr+min temp) | 1403.69 | 0.62 | 0.1332 | 0.7334 | 8 | 1387.69 |
| ψ(DEM^2*NDVI^2),ρ(yr+min temp) | 1403.99 | 0.92 | 0.1146 | 0.6313 | 9 | 1385.99 |
| ψ(DEM^2),ρ(yr+min temp) | 1404.01 | 0.94 | 0.1135 | 0.6250 | 7 | 1390.01 |
| ψ(DEM^2*feed trees),ρ(yr+min temp) | 1405.06 | 1.99 | 0.0671 | 0.3697 | 9 | 1387.06 |
| ψ(DEM^2*fire),ρ(yr+min temp) | 1405.69 | 2.62 | 0.0490 | 0.2698 | 9 | 1387.69 |
| ψ(DEM^2+recent),ρ(yr+min temp) | 1405.76 | 2.69 | 0.0473 | 0.2605 | 8 | 1389.76 |
| ψ(DEM^2+cleared),ρ(yr+min temp) | 1405.95 | 2.88 | 0.0430 | 0.2369 | 8 | 1389.95 |
| ψ(DEM^2+heavy),ρ(yr+min temp) | 1405.96 | 2.89 | 0.0428 | 0.2357 | 8 | 1389.96 |
| ψ(DEM^2+old growth),ρ(yr+min temp) | 1405.96 | 2.89 | 0.0428 | 0.2357 | 8 | 1389.96 |
| ψ(DEM^2+trt),ρ(yr+min temp) | 1413.66 | 10.59 | 0.0009 | 0.0050 | 15 | 1383.66 |
| ψ(NDVI^2+feed trees),ρ(yr+min temp) | 1415.41 | 12.34 | 0.0004 | 0.0021 | 8 | 1399.41 |
| ψ(NDVI^2),ρ(yr+min temp) | 1416.10 | 13.03 | 0.0003 | 0.0015 | 7 | 1402.1 |
| ψ(feed trees),ρ(yr+min temp) | 1416.46 | 13.39 | 0.0002 | 0.0012 | 7 | 1402.46 |
| ψ(global),ρ(yr+min temp) | 1417.58 | 14.51 | 0.0001 | 0.0007 | 21 | 1375.58 |
| ψ(NDVI^2+fire),ρ(yr+min temp) | 1417.78 | 14.71 | 0.0001 | 0.0006 | 8 | 1401.78 |
| ψ(NDVI^2+heavy),ρ(yr+min temp) | 1417.78 | 14.71 | 0.0001 | 0.0006 | 8 | 1401.78 |
| ψ(NDVI^2+old growth),ρ(yr+min temp) | 1417.79 | 14.72 | 0.0001 | 0.0006 | 8 | 1401.79 |
| ψ(NDVI^2+cleared),ρ(yr+min temp) | 1417.96 | 14.89 | 0.0001 | 0.0006 | 8 | 1401.96 |
| ψ(NDVI^2+trt),ρ(yr+min temp) | 1419.97 | 16.90 | 0.0000 | 0.0002 | 15 | 1389.97 |
| ψ(.),ρ(yr+min temp) | 1419.98 | 16.91 | 0.0000 | 0.0002 | 6 | 1407.98 |
| ψ(fire),ρ(yr+min temp) | 1421.36 | 18.29 | 0.0000 | 0.0001 | 7 | 1407.36 |
| ψ(old growth),ρ(yr+min temp) | 1421.81 | 18.74 | 0.0000 | 0.0001 | 7 | 1407.81 |
| ψ(cleared),ρ(yr+min temp) | 1421.86 | 18.79 | 0.0000 | 0.0001 | 7 | 1407.86 |
| ψ(heavy),ρ(yr+min temp) | 1421.86 | 18.79 | 0.0000 | 0.0001 | 7 | 1407.86 |
| ψ(recent),ρ(yr+min temp) | 1421.92 | 18.85 | 0.0000 | 0.0001 | 7 | 1407.92 |
| ψ(feed trees+trt),ρ(yr+min temp) | 1422.72 | 19.65 | 0.0000 | 0.0001 | 15 | 1392.72 |
| ψ(NDVI^2*trt),ρ(yr+min temp) | 1424.50 | 21.43 | 0.0000 | 0.0000 | 23 | 1378.5 |
| ψ(feed trees*trt),ρ(yr+min temp) | 1425.39 | 22.32 | 0.0000 | 0.0000 | 23 | 1379.39 |
| ψ(trt),ρ(yr+min temp) | 1425.67 | 22.60 | 0.0000 | 0.0000 | 14 | 1397.67 |
| ψ(DEM^2*trt),ρ(yr+min temp) | 1425.82 | 22.75 | 0.0000 | 0.0000 | 23 | 1379.82 |
| ψ(trt+fire),ρ(yr+min temp) | 1426.99 | 23.92 | 0.0000 | 0.0000 | 15 | 1396.99 |
| ψ(trt+cleared),ρ(yr+min temp) | 1427.21 | 24.14 | 0.0000 | 0.0000 | 15 | 1397.21 |
| ψ(trt*fire),ρ(yr+min temp) | 1433.27 | 30.20 | 0.0000 | 0.0000 | 23 | 1387.27 |

Light grey shading indicates supported models for detectability. Estimates for occupancy were model averaged across supported models.
